# How frequently external ventricular drainage device should be changed in children with ventriculoperitonel shunt infection?

**DOI:** 10.12669/pjms.312.6515

**Published:** 2015

**Authors:** Ismail Gulsen, Hakan Ak, Nihat Demir, Enver Sosuncu, Mehmet Arslan

**Affiliations:** 1 Ismail Gulsen, Department of Neurosurgery, Yuzuncu Yil University, School of Medicine, Van, Turkey; 2 Hakan Ak, Department of Neurosurgery, Bozok University, School of Medicine,Yozgat, Turkey; 3 Nihat Demir, Department of Pediatrics, Yuzuncu Yil University, School of Medicine, Van, Turkey; 4 Enver Sosuncu, Department of Neurosurgery, Yuzuncu Yil University, School of Medicine, Van, Turkey; 5 Mehmet Arslan, Department of Neurosurgery, Yuzuncu Yil University, School of Medicine, Van, Turkey

**Keywords:** Hydrocephalus, Ventriculoperitoneal shunt infection, External ventricular drainage

## Abstract

**Objective:**

The purpose of the presenting study was to determine how frequently external ventricular drainage (EVD) device should be changed in children with ventriculopertienal shunt (VPS) infection during prolonged intravenous antimicrobial therapy.

**Methods:**

In this retrospective study, 25 children with VPS infection were evaluated between January 2012 and December 2013. In these children VPS was surgically removed and appropriate antimicrobial therapy was administered according to cerebrospinal culture results. Data noted about how frequently EVD device had been changed, the number of cells on direct observation of cerebrospinal fluid (CSF), glucose and protein levels of CSF, and CSF culture results were obtained from patients’ records.

**Results:**

Total 25 children were included in the study. The median age was three months (1 and 65 months). In 44% of children, Staphylococcus epidermidis was isolated. During treatment period, EVD catheter has changed one to six times. A total of 68 EVD catheters were changed in these patients. When the duration of ventriculostomy catheter and leukocyte count in CSF were evaluated on daily basis, leukocyte count was decreased 5 units per day in children whose catheter remained less than 10 days. However, in children whose catheter remained more than 10 days leukocyte count was decreased 2.21 units per day.

**Conclusions:**

In children with VPS infection, EVD device should be changed at every 10 days for the rapid resolution of the infection.

## INTRODUCTION

Hydrocephalus is a common pathology in daily practice of neurosurgery and the widely accepted approach in the treatment of this pathology is the insertion of a ventriculoperitoneal shunt (VPS). VPS application may be performed nearly in all neurosurgery clinics. However, it has its own complications both in preoperative and postoperative periods. One of the most common complication is the ventriculoperitoneal shunt infection which is an important cause of morbidity and mortality in affected children.^1^-^3^ The incidence of shunt infection varies between 5-10%.^4^,^5^

In the management of infective cases, the preferred treatment strategy is the surgical removal of shunt, administration of antimicrobial therapy, installation of an external ventricular drainage device, and placement of a new shunt when CSF become sterile.^6^ Although it is an established procedure, the frequency of EVD replacement during prolonged antibiotherapy has not defined clearly in the literature.

In the presenting retrospective study, we aimed to describe how frequently EVD should be changed until CSF becomes sterilized.

## METHODS

This retrospective study (Noninvasive Ethics Committee of Yuzuncu Yil University School of Medicine at its meeting of 30 January 2014 the number 10) included 25 children who were hospitalized due to ventriculoperitoenal shunt infection in Yuzuncu Yil University Hospital between January 2012 and December 2013. Data about the patients were obtained from the hospital recordings. All of these children had communicating hydrocephalus. 5 children with inadequate data, 8 children with non-communicating hydrocephalus and 2 children who were failed during treatment due to sepsis were excluded from the study.

In our clinic as a routine practice, the diagnosis of VP shunt infection is performed with the patient’s complaints (headache, nausea, vomiting and seizure), medical history and physical examination findings (fever, changes in level of consciousness, wound discharge, neck stiffness, and Kerning and Brudzinsky’s signs), and laboratory findings (view of CSF, cell number, cell type, protein and glucose levels, and culture) as in the literature.^4^,^5^,^7^

Patients diagnosed with shunt infection were operated in 6 hour of admission under local or general anesthesia according to the general status of the child. In the operation VP shunt surgically removed and EVD device was installed from right Kocher’s point. After that intravenous antibiotic therapy was started according to CSF culture results.

Until CSF become sterilized, data noted about how frequently EVD had been changed, the number of cells, glucose and protein levels, and culture of CSF at every change were noted.

### Installation of the Pressure Controlled Ventricular Drainage Catheter

Patient was positioned supine with thirty degrees flexion to neck and shoulder. A frontal burr-hole (2 to 3 cm lateral to midline and 1cm anterior to coronal sutura in the midpupillary line = Kocher’s point) was opened. Ventricular catheter was placed into the frontal horn of same sided lateral ventricle. CSF drainage system was connected to the manometer after observing CSF flow. Drainage pressure was adjusted to be 10 to 15 cm H2O. This pressure was achieved by elevating the drainage catheter above 10-15 cm of foramen Monro. CSF samples were taken periodically every 3 days and glucose, protein, and cell counts were performed. Daily CSF volume and appearance (such as clear or xanthochromic) were noted. All of these applications are performed in our clinic as a part of routine process in patients presenting with shunt infection.

## RESULTS

Total 25 children were included in the study. The median age of them was three months (ranging between one month and 65 months). Thirteen of them were female (52%) and 12 were male (48%). In 11 (44%) children, S. Epidermidis was isolated in culture. Results of CFF culture are demonstrated Table-I.

**Table-I:** Causative agents in infected children.

Causative agent	Frequency	Percent
Staf epidermidis	11	44.0
Klebsiella Pneu.	3	12.0
Strep. Pneum	3	12.0
Enterococous faecium	3	12.0
E.Coli	2	8.0
Methcillin resistant staf. auereus	2	8.0
Psödomanas	1	4.0
Total	25	100.0

We detected that in the treatment of children whose CSF culture results revealed gram-positive microorganisms, vancomycin, rifampicin, linezolid were used. In the management of children with gram-negative bacterial growth in culture, meropenem, ceftriaxone, cefaperazone-sulbactam were used in according to the consultation of pediatric infectious diseases.

During treatment period, EVD catheter has changed one to six times. Totally, 68 times EVD catheter was changed (Table-II). The average duration of a ventriculostomy catheter was 12.62±4.10 days. 19 (30%) of them were stayed less than 10 days. However, 47 (70%) of them were stayed more than 10 days.

**Table-II:** EVD change numbers.

No. of EVD changing	No. of patients	Percent
1	4	16,0
2	9	36,0
3	5	20,0
4	5	20,0
5	1	4,0
6	1	4,0
Total	25	100,0

When duration of a ventriculostomy catheter and leukocyte count in CSF were evaluated on a daily basis, we saw that leukocyte count decreased 5 units per day in children whose catheter remained less than 10 days. However, in children whose catheter remained more than 10 days leukocyte count decreased 21.2 units per day. In the detection of daily decrease of leukocyte count, leukocyte count difference between on the day of insertion of the EVD catheter and on the day of removal of the catheter divided to length of stay of EVD catheter (Grap.1).

**Grap.1:**
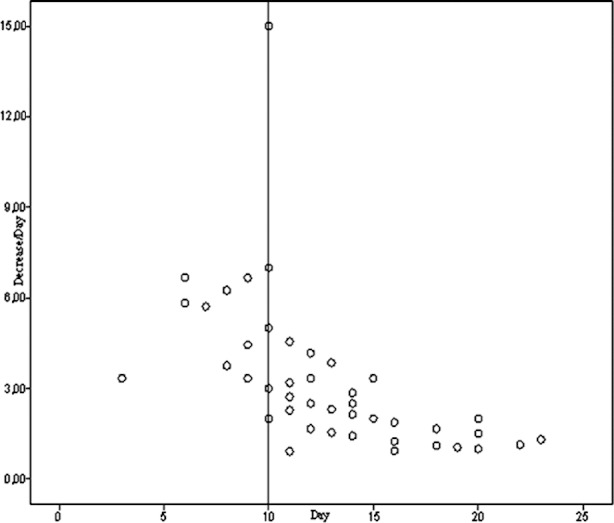
The relationship between decrease of leucocyte count and day.

### Statistical Analysis

Descriptive statistics for studied variables (characteristics) were presented as median mean, standard deviation, minimum and maximum values. Mann- Whitney U test was used to compare groups’ median for the studied variables. Statistical significance levels were considered as 5%. The SPSS (ver. 13) statistical program was used for all statistical computation (Table-III).

## DISCUSSION

With the advent of valve shunt system in 1949 by Nulsen and Spitzer, a revolution has been experienced in the management of hydrocephalus.^1^ Despite the constantly evolving shunt technology, about 70% of patients who are inserted ventriculoperitoneal shunt are faced with shunt dysfunction within ten years.^8^ The most common complications are the mechanical shunt obstruction and shunt infection.^9^-^13^

**Table-III:** Statistical results of blood and cerebrospinal fluid.

	0-10 (day)					11-50 (day)					
	Median	Mean	Std. dev	Min	Max.	Median	Mean	Std. dev.	Min.	Max.	p
CSF protein	160,00	291,68	364,92	45,00	1539,00	210,00	351,83	362,90	19,00	2039,00	0.54
CSF glucose	25,00	26,47	19,39	1,00	65,00	23,00	26,48	16,69	0.45	84,00	0.99
CRP	45,00	52,60	61,19	3,00	154,00	55,00	50,43	39,73	3,00	120,00	0.92
WBC in blood	18,00	19,00	9,27	9,00	35,00	12,70	12,87	3,99	6,00	22,00	0.59
CSF leucocyte	50,00	84,41	92,02	10,00	400,00	65,00	68,15	33,54	10,00	150,00	0.30
Leucocyte difference	40,00	46,18	30,49	10,00	150,00	30,00	29,78	9,83	10,00	50,00	0.002
Leucocyte decrease/day	5,00	5,45	2,89	2,00	15,00	2,21	2,20	0.94	0.91	4,55	0.001

Yogev et al. reported that 50% of shunt infections developed within the two weeks after operation and causative agent was the Staphylococcus epidermidis in 60% of cases.^13^ In a different study, Telhan et al. reported the causative agent Staphylococcus epidermidis and staf aures in 30% of cases.^11^ In our study, the most common causative agent was the Staphylococcus epidermidis in 44% of cases (Table-I). The most probable cause of this causative agent is the contamination from the skin during surgical procedure.^9^,^12^

There are three different approaches when faced with VP shunt infection. These are; giving intravenous antibiotic therapy without removing shunt infected shunt, giving antibiotic regiment after surgical removal of shunt, and installation of EVD after removal of shunt and giving antibiotic regiment.^10^,^12^,^14^,^15^ Schreffler et al. compared these three methods and they concluded that the best result was gained with antibiotic regiment after the removal of infected shunt with establishment of EVD.^16^ In our clinic, we also perform the same approach composed of removal of infected shunt with establishment of EVD and giving antibiotic regiment according to culture results. Literature does not contain definite information about how frequently EVD should be changed in infected cases. In our study we detected that EVD had been changed in 6 to 22 days (mean 12.62 ± 4.10 days).

On the literature search, we found that in non-infected cases with ventricular hemorrhage authors reported the relationship between the development of infection and drainage change frequency. Wong et al. found that regular changes of ventricular catheter at five day intervals did not reduce the risk of CSF infection. They also stated that a single external ventricular drain can be employed for as long as clinically indicated.^17^ However, in another study Kiymaz et al. reported that changing the EVD catheter on each 6 days didn’t cause infection in patients with intraventicular hemorrhage.^18^ Holloway et al.^19^ evaluated the effect of monitoring duration and catheter exchange and their relationship about the ventriculostomy infection in their restrospective study including 584 cases. They stated that external ventricular drainage application increases the risk of infection until 10 days, however, there was no increased risk after 10 days. They concluded that ventriculostomy catheter for intracranial pressure monitoring should be removed as quickly as possible. According to our opinion their most striking conclusion was that in cases requiring prolonged monitoring, catheter exchange had no benefit on risk of ventriculostomy infection.^19^ However, in all of the three studies mentioned above, there was no infection at the presentation of the patients.

Our study directly related with the infected cases. We think that infective agent may colonize throughout or at anywhere of the catheter. In our study, patients were divided into two groups; drainage with EVD less than 10 days and drainage with EVD more than 10 days. Average leukocyte decrease in CSF was 5 leukocytes/day (2-15 leukocyte/day) in patients who drained less than 10 days. However, this ratio was 2.21 leukocyte/day (0.91-4.55 leukocyte/day) in patients drained more than 10 days (Grap.1). Statistically significant difference was found between two groups (p=0.02). According to this result we suggest that in patients whose EVD stayed more 10 days, resolution rate of infection decreases and the antibiotic response to infection also decreases.

### Limitations

There are two main limitations of our study. First one of them is the retrospective nature of our study and second one is the small number of our study group.

## CONCLUSIONS

In children with VP shunt infection, infected shunt should be removed and external ventricular drainage should be inserted. Additionally, for the rapid resolution of infection EVD should be changed at every 10 days independently from the causative agent. However, future prospective multicentered studies containing lager number of patients is needed to prove our results.
